# Tunable Construction of Chiral Nematic Cellulose Nanocrystals/ZnO Films for Ultra-Sensitive, Recyclable Sensing of Humidity and Ethanol

**DOI:** 10.3390/ijms25094978

**Published:** 2024-05-02

**Authors:** Xiao Xiao, Hanqi Dong, Xinxin Ping, Guowei Shan, Jie Chen, Mengxing Yan, Weixing Li, Zhe Ling

**Affiliations:** 1 State Key Laboratory of Materials-Oriented Chemical Engineering, College of Chemical Engineering, Nanjing Tech University, Nanjing 211816, China; 2 Jiangsu Co-Innovation Center of Efficient Processing and Utilization of Forest Resources, College of Chemical Engineering, Nanjing Forestry University, Nanjing 210037, China

**Keywords:** cellulose nanocrystals, iridescent nanocomposite films, chiral nematic structure, environment-responsive sensor, ZnO nanoparticles

## Abstract

The investigation of functional materials derived from sustainable and eco-friendly bioresources has generated significant attention. Herein, nanocomposite films based on chiral nematic cellulose crystals (CNCs) were developed by incorporating xylose and biocompatible ZnO nanoparticles (NPs) via evaporation-induced self-assembly (EISA). The nanocomposite films exhibited iridescent color changes that corresponded to the birefringence phenomenon under polarized light, which was attributed to the formation of cholesteric structures. ZnO nanoparticles were proved to successfully adjust the helical pitches of the chiral arrangements of the CNCs, resulting in tunable optical light with shifted wavelength bands. Furthermore, the nanocomposite films showed fast humidity and ethanol stimuli response properties, exhibiting the potential of stimuli sensors of the CNC-based sustainable materials.

## 1. Introduction

As the most abundant and renewable biopolymer on earth, cellulose has achieved considerable interest in preparing functional materials [1,2,3,4,5]. Cellulose fibers, commonly derived from trees, plants, fungi, and bacteria, are composed of repeated β-1,4-linked glucosidic units. The aggregation of the glucan chains of cellulose can form a distinctive two-phase structure, which contains both a well-aligned crystalline part and a disordered amorphous part [6]. Acid or enzymatic hydrolysis treatments on cellulose chains may effectively remove the amorphous region and retain the crystalline domains, resulting in rigid and rod-like cellulose nanocrystals (CNCs) [7,8]. Nowadays, CNCs have attracted significant research interest due to the high stiffness, low density, chemical modification potential, and colloidal stability caused by the negatively charged sulfate groups on the surface of CNCs [9]. 

One of the most attractive applications of CNCs is the liquid crystal phase formed in its colloid suspension [10]. Further, the chiral nematic structure present in the suspension can be well-preserved via evaporation-induced self-assembly (EISA). The resulting chiral nematic CNC films selectively reflect left-handed circularly polarized light, performing birefringence under POM and structural color under visible light [11]. The iridescence of the CNC films via EISA relies on the reflected wavelength, which is mainly according to the helical pitch of the periodic structures. The pitch value was reported to be affected by a series of factors [12]. Firstly, the ionic strength of the CNC suspension is a critical factor, which showed a negative effect on helical pitch values [13]. Moreover, the chiral arrangements of the CNCs were highly sensitive to environmental changes, such as humidity, temperature, acidity, outer stress, and other chemical adsorption, which offered promising opportunities for fabricating environmental-responsive materials [14,15,16,17,18].

Intercalation of other chemicals, such as polysaccharides, was reported as an effective way to tune the helical pitches and optical iridescence of the EISA CNC films [19]. Also, the mechanical strength of the films was significantly improved due to the increasing amount of hydrogen bonding linkages, which resolved the problems of brittleness and low flexibility. In addition, metallic nanomaterials have been shown to have distinctive optical properties due to unique size effects [20]. Various kinds of metals and metal oxides, including silver, gold, copper, iron, and zinc nanoparticles, were introduced to chiral nematic CNCs not only to improve the optical properties but also to bring in other new functions to the materials [21]. By introducing and controlling the density and dimensions of gold nanoparticles, chiral nematic CNC films achieved a two-fold enhancement of the polarization rotation power, which proved the advantages of metals in designing functional CNC materials [22]. The cholesteric arrangements of the rod-like nanoparticles were reported to be controlled by the magnetic fields. By using commercial metal compounds neodymium (NdFeB), solid nanostructured films were produced with improved uniformity in the orientation of the cholesteric axes, leading to large homogenous films and better control over their orientation [23]. Further, a simpler approach to tuning CNC arrangements was achieved by applying an ultrasmall magnetic field using Fe_3_O_4_ nanoparticles [24]. The helical pitches were found to drop with an increase in the applied magnetic field. The research provides a new strategy for combining Chiral CNC materials with metal oxides to enable varying applications. Recently, the co-assembly behavior of high-loading TiO_2_ nanorods with CNCs in suspension was researched through water removal and calcination, indicating an approach of universal application of ligand-stabilized inorganic nanorods [25]. 

ZnO is a typical metal oxide with a size range from 2 to 7 nm, and it has excellent electrical and mechanical properties [26]. Metal oxide was generally applied to antifouling and antibacterial materials fabrication, as well as photocatalytic fields [27]. CNCs-ZnO nanocomposite films have been reported for over a decade, which greatly improved the applicable properties like mechanical strength, thermal stability, UV-shielding properties, and antimicrobial capability [28]. In terms of optical properties, a luminescent ZnO quantum dot/CNCs nanohybrid ink was produced via electrostatic self-assembly for solvent resistance and message encryption process, which furtherly widened the application of CNCs-ZnO nanocomposite materials [29]. However, the incorporation of ZnO nanoparticles with chiral nematic CNC films has been rarely reported, and the application of the iridescence phenomenon of CNCs in the presence of ZnO urgently needs to be investigated.

In this work, ZnO nanoparticles were introduced to the cholesteric structure of CNCs, and the chiral nematic nanocomposite films were fabricated via EISA. The chemical, morphological, and crystal structures of the materials were determined. Moreover, the application of the materials to reversible environmental changes such as humid and ethanol responses was proposed, which may expand the application strategy of CNC chiral nematic films in the fields of environmental protection, wearable devices, food packaging, etc.

## 2. Results and Discussion

### 2.1. Optical Properties of the CNC/Xylose/ZnO Nanocomposite Films

**Figure 1:**
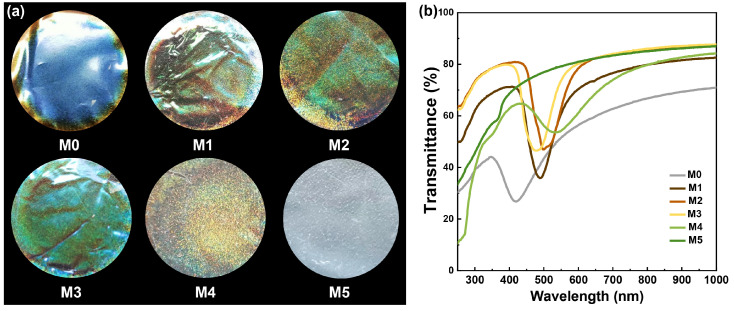
(**a**) Optical images and (**b**) UV–vis spectra of different samples.

The photographs of nanocomposite films are shown in Figure 1, revealing a noticeable color change in the films. The pristine CNC/xylose film exhibited a dark blue color, while the addition of ZnO NPs resulted in a redshift in iridescence. Subsequently, the green color of M2 and M3 gradually changed to the orange shade of M4, ultimately changing to light gray, indicating that the chiral nematic structure of rod-like CNC nanoparticles was disturbed (Figure 1a). Moreover, similar changes occurred at the edge of the circular film in M0 and M1 due to the “coffee ring” effect. Furthermore, as the concentration of ZnO NPs increases, a shift from dark blue to uniform yellow and light gray colors is observed in the film. It was evident that this led to the limited manifestation of the resulting “coffee ring” effect but maintained a uniform yellow appearance. UV–vis tests further confirmed both the optical properties and iridescent coloration of the nanocomposite films (Figure 1b). The films demonstrated high light transmittance within the visible light range, substantiating the excellent transparency property. Each film displayed distinct characteristic absorption peaks indicating alterations in iridescent coloration trends. Notably, M0 exhibited over 60% transmittance with its characteristic absorption peak around 420 nm. With an increase in ZnO NP content, a redshift was observed for characteristic absorption peaks of each film, suggesting a transition from dark blue towards an orange-red region consistent with iridescent changes shown in Figure 1a. It was worth noting that no prominent maximum absorption peak was observed for M5, signifying damage to its chiral helix nematic structures, leading to the disappearance of the iridescence aligning with the optical observations.

**Figure 2:**
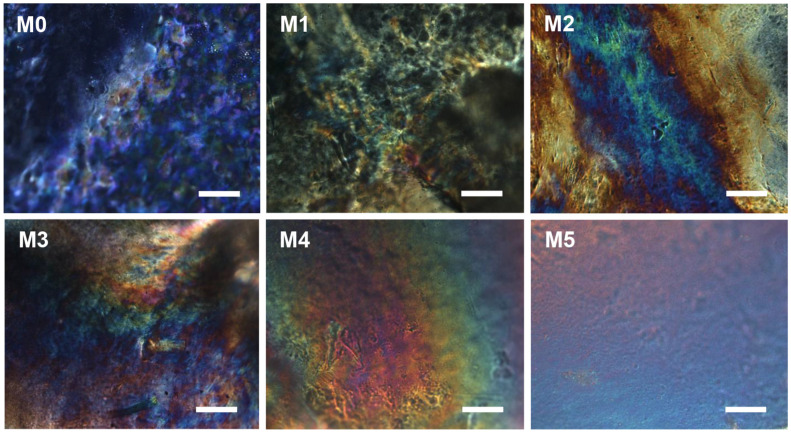
POM images for different samples. Scale bar = 100 μm.

Furthermore, the birefringence phenomenon of the films was observed under the polarized light in Figure 2, revealing a typical finger texture structure that indicated the formation of a characteristic chiral nematic phase structure. With the increase in ZnO NP concentration, a clear rainbow finger texture emerged in M3 and M4, while this effect was less pronounced in M5, suggesting that the chiral nematic structure can be tuned by varying the containing of ZnO NPs.

### 2.2. Morphology of the CNC/Xylose/ZnO Nanocomposite Films

**Figure 3:**
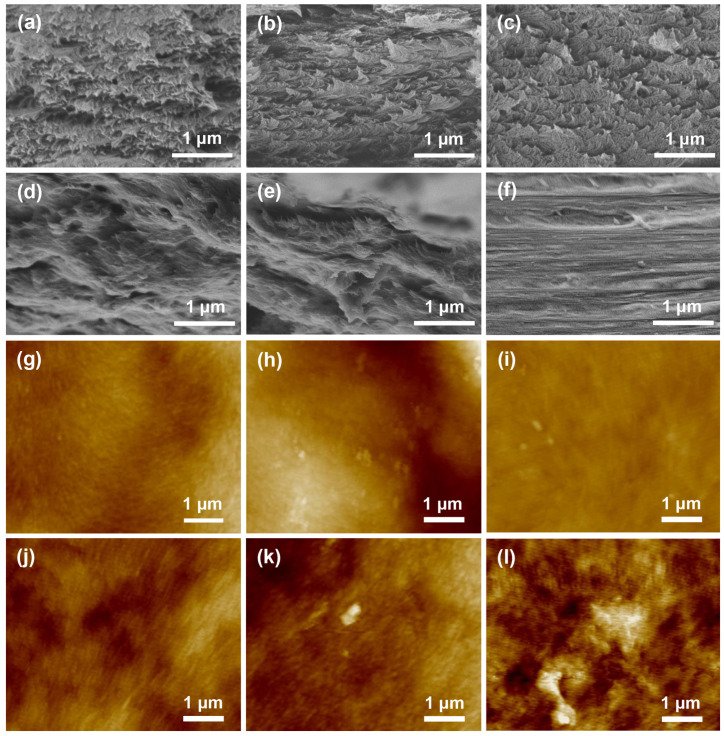
SEM images (**a**–**f**) and AFM images (**g**–**l**) for M0 (**a**,**g**), M1 (**b**,**h**), M2 (**c**,**i**), M3 (**d**,**j**), M4 (**e**,**k**), and M5 (**f**,**l**).

The chiral nematic arrangements of the nanocomposite films were clearly observed by SEM in Figure 3a–f. Cross-sectional observation revealed the presence of a multiple-layered structure in the pristine CNC/xylose (M0) nanocomposite film. The complete spiral structure was formed through the orderly arrangement of rod-like crystals, indicating the chiral nematic structure formed by the induction of self-assembly during evaporation. When the concentration of the ZnO NPs increased, an enlarged helical pitch was observed. Correspondingly, there was a redshift phenomenon in the reflected color of macroscopic iridescent films, consistent with the optical observation in Figure 1a. It was worth noting that no obvious cholesteric spiral structures were observed in the M5 film. This further confirmed that the excessive addition of ZnO NPs affected the chiral nematic structure, resulting in a weakened iridescent structural color of M5 film compared to other films, which aligned with the optical observation and UV–vis spectra. The surface structure was examined by AFM (Figure 3g–l), which showed that the regular rod-like nanocrystal structures were consistent with the chiral nematic structure seen in SEM cross-section images. With the increasing addition of ZnO NPs, nanoparticles were evenly dispersed on the surface of the films, particularly evident in M3 and M4. The significant increase in nanoparticle density, along with an increase in array structure gap, affected the nematic structure of the cholesteric phase, which corresponded to the wavelength redshift phenomenon observed via UV–vis spectra.

### 2.3. Crystal and Chemical Structures of the CNC/Xylose/ZnO Nanocomposite Films

**Figure 4:**
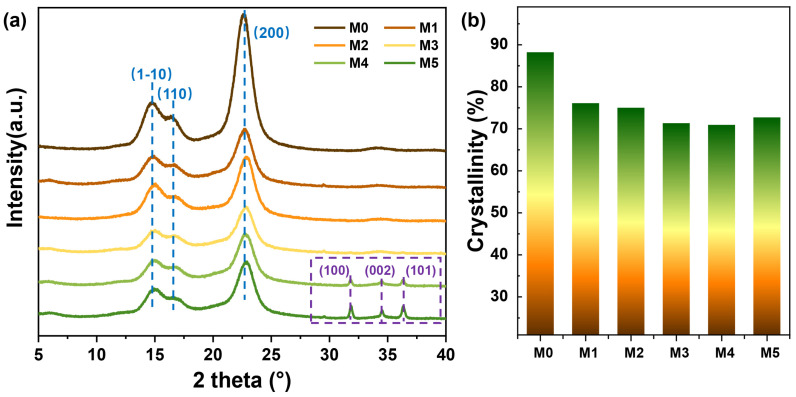
(**a**) XRD spectra and (**b**) crystallinity for different films.

The nematic arrangement and crystal structures of the nanocomposite films were confirmed by XRD analysis in Figure 4. The typical peaks at 14.8°, 16.5°, and 22.5° were assigned to (1–10), (110), and (200) lattice planes of cellulose, respectively. The intense and narrow peak corresponding to the (200) lattice planes in the M0 film revealed high crystallinity (~88.2%), consistent with the SEM observations. The non-uniform peak heights observed for the hydrophilic lattice surfaces (1–10) and (110) were primarily attributed to the anisotropic orientation structure of cellulose crystals, particularly induced by the EISA membrane cholesteric phase structure [30]. This structure favored the formation of chiral nematic films, resulting in a noticeable iridescent phenomenon. With the addition of xylose, there was a decrease in crystallinity mainly due to an amorphous structure of xylose, which increased the proportion of amorphous regions and reduced the overall crystallinity of the films. The crystallinity decreased to approximately 70% for M3, M4, and M5 films, indicating an increase in the material flexibility after composite modification that facilitated its macro utilization. Further analysis revealed distinct spikes at 2*θ* = 32.1°, 34.5° and 36.8° for the M4 and M5 films as ZnO NP content increased gradually, representing diffraction peaks corresponding to ZnO crystal planes at (100), (002) and (101), respectively [31]. This phenomenon indicated that the deposition and distribution of ZnO NPs on the nanocomposite films inducing recrystallization occurred primarily along the vertical direction of the cellulose crystal chain, thereby facilitating regulation of pitch for the chiral arrangement of CNC rod-like crystals within the films.

**Figure 5:**
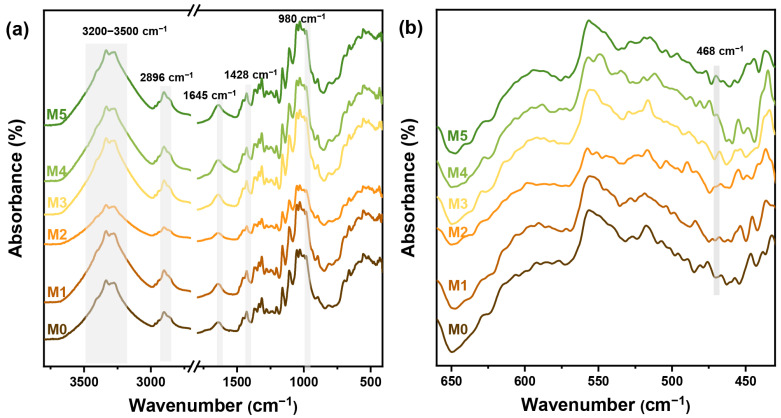
FT-IR spectra of different samples (**a**) and the enlarged wavenumber ranges of 425 cm^−1^–660 cm^−1^ (**b**).

The chemical structure of the composite film was investigated by FT-IR analysis, as shown in Figure 5. The peak observed at 2896 cm^−1^ corresponded to the stretching vibration of C-H bonds, and peaks at 1428 cm^−1^ and 1369 cm^−1^ corresponded to the bending vibration of C-H bonds. Additionally, the peak observed at 980 cm^−1^ was attributed to the stretching vibration of C-O-C. These characteristic peaks were indicative of cellulose and polysaccharide samples present in the films. The wide peak observed between 3200 cm^−1^ and 3500 cm^−1^ represented hydrogen bonds formed within both intermolecular and intramolecular hydroxyl groups in polysaccharides (Figure 5a). With the addition of Xylose and ZnO NPs with varying concentrations, an increase in peak width was observed, indicating a higher abundance of hydrogen bonds. Therefore, the characteristic peaks of cellulose and polysaccharides suggested that the addition of ZnO NPs had no effect on the chiral nematic structure of the cellulose skeleton in nanocomposite films. The antisymmetric stretching peak corresponding to C=O was detected at 1645 cm^−1^ due to the galacturonic acid group introduced after the addition of xylose, which proved the uniform distribution of xylose in the nanocomposite films. Furthermore, a new absorption peak corresponding to Zn-O stretching was detected at 468 cm^−1^ [32], confirming the successful incorporation of ZnO NPs into the nanocomposite film and the disruption of the crystallinity of the composite materials (Figure 5b) [33].

**Figure 6:**
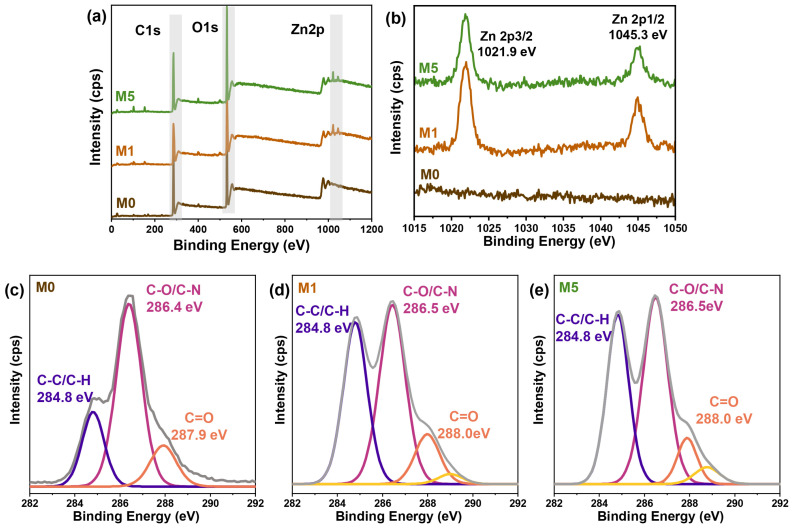
XPS spectra of the M0, M1, and M5 films. (**a**) Full survey scan, (**b**) Zn 2p spectra, and C1s spectra of the (**c**) M0, (**d**) M1, and (**e**) M5 films.

The chemical composition of nanocomposite films was further analyzed by XPS (Figure 6). The wide scan spectra were presented in Figure 6a, where remarkable peaks corresponding to C1s and O1s can be observed in the M0, M1, and M5 films. With the addition of ZnO NPs, new peaks were observed in the spectra of the M1 and M5 films. Enlarged new peak spectra were shown in Figure 6b, with characteristic peaks at 1021.9 eV and 1045.3 eV ascribed to Zn 2p3/2 and Zn 2p1/2, respectively. Additionally, high-resolution spectra of C1s were shown in Figure 6c–e. It was found that the highest peak strength of C-O at 286.4 eV was observed in the M0 film (Figure 6c), primarily due to the presence of glucan molecules within the CNC skeleton connected by representative β-1,4-glucoside bonds. With the introduction of Xylose and ZnO NPs, there were no significant changes observed in the C-O bond, and the content of the C-O bond remained high, indicating it as a predominant chemical bonding bond within nanocomposite thin films. Notably, there was a remarkable increase in characteristic peaks related to C-C/C-H bonds at 284.8 eV for both the M1 and M5 films (Figure 6d,e) due to a higher content of C-C/C-H bonds present within Xylose molecular chains. These results contributed towards an increased formation of hydrogen bonds within the nanocomposite films while enhancing the flexibility and mechanical strength of the films.

### 2.4. Environmental Responses of the CNC/Xylose/ZnO Nanocomposite Films

**Figure 7:**
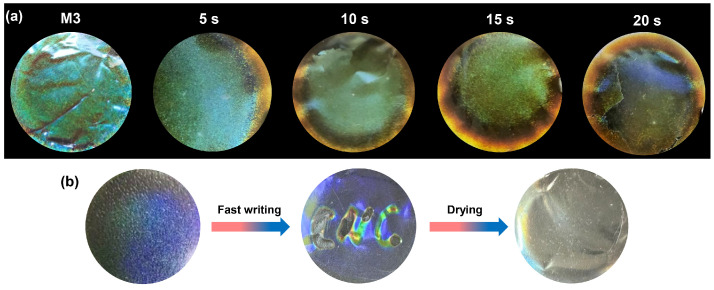
(**a**) Optical iridescence changes in the M3 film under the humid environment at different time points. (**b**) The recycling of water writing and drying of the films.

The nanocomposite films exhibited a rapid and reversible response to humidity, as shown in Figure 7. When the films were exposed to vapor, an immediate color change was observed, characterized by a redshift in iridescence. The film transformed into a yellow hue with a remarkable “coffee ring” effect within 20 s, which can be attributed to the asymmetrical drying flow during film formation. Leveraging this fast vapor response, we successfully achieved rapid writing and drying recovery cycles of the films (Figure 7b).

**Figure 8:**
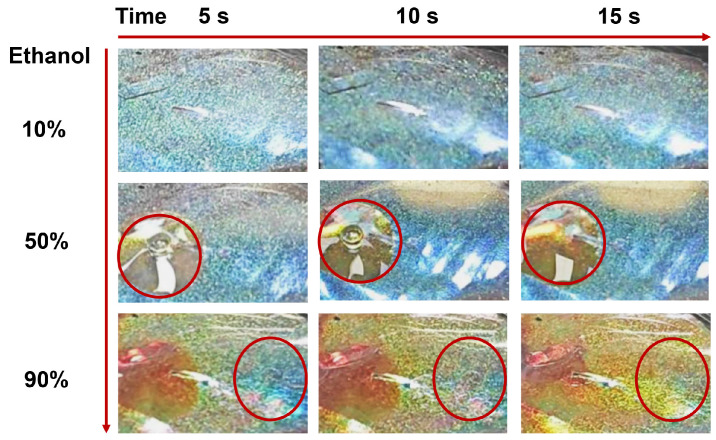
Optical iridescent changes (red circles refer to the same region) in the M2 film under the different concentrations of ethanol.

Furthermore, the responsiveness of the nanocomposite films to ethanol was observed (Figure 8). The films were cut into long strips, and a drop of aqueous ethanol solution with varying concentrations was added to one end while color changes were observed at the other end of the strip. It was found that a 10% ethanol solution did not affect the iridescence of the film. When 50% ethanol was added, there was no color change at the remote end, while a redshift in iridescence occurred at the position where 50% ethanol solution was added. Notably, exposure to a 90% ethanol aqueous solution caused a blue-to-orange color change with significant redshift at the remote end and red coloring where drops were added. These findings demonstrated that nanocomposite films displayed the potential as sensors for detecting different concentrations of ethanol.

**Figure 9:**
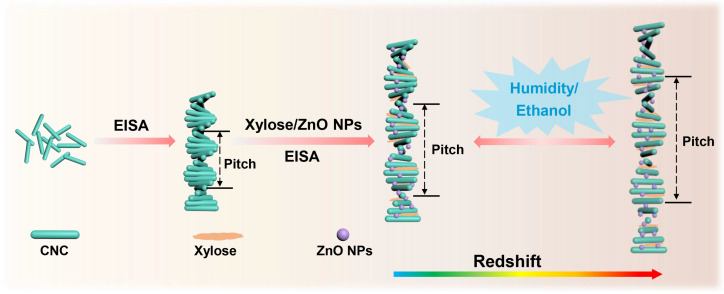
Schematic illustration of the tunable of the proposed CNC chiral nematic structures by the addition of xylose and ZnO NPs.

The schematic illustrated in Figure 9 depicts the mechanism of the responsive iridescent change. The chiral nematic structure was formed through EISA by CNCs, which can be regulated by the addition of xylose and ZnO NPs. Specifically, the introduction of molecules and nanoparticles increased the helical pitch, resulting in a redshift in optical iridescence observed in the films. As environmental humidity rose, the optical iridescence turned orange, indicating the intercalation of water molecules that led to an increase in helical pitch. Similarly, ethanol molecules regulated the pitch adjustment, causing a color redshift response in the films.

## 3. Materials and Methods

### 3.1. Materials

CNC suspension was prepared by the hydrolysis of softwood bleaching pulp (Huatai Paper Co., Ltd., Dongying, Shandong, China) in a H_2_SO_4_ solution with a concentration of 60% at 55 °C for 75 min, which was stirred at 400 rpm for 1.5 h and finalized by the addition of deionized water. The obtained suspension was washed with deionized water and centrifuged at 10,000 rpm for 15 min to remove extra acid. Then, the suspension was dialyzed with deionized water for 7 days until the pH reached around 6. Finally, the CNC suspension was concentrated at 3% (*w*/*v*). Xylose (AR) and ZnO nanoparticles (AR) were purchased from Macklin Chemical Co., Ltd., Shanghai, China.

### 3.2. Fabrication of CNC/Xylose/ZnO Nanocomposite Films

First, a certain amount of xylose powder and ZnO NPs was weighed into a CNC suspension with mechanical stirring to prepare the mixed suspensions. The solids ratios of CNC, xylose, and ZnO NPs were 100:20:0, 100:20:1, 100:20:5, 100:20:10, 100:20:15, and 100:20:20, respectively (Table 1). The mixed suspensions were stirred at room temperature for 6 h. Subsequently, the obtained six groups of homogeneous suspensions were cast in Petri dishes with a diameter of 30 mm and evaporated at room temperature for 4–7 days to form the nanocomposite films. The films were marked as M0–M5, respectively, as shown in Table 1.

### 3.3. Characterizations

The optical transmittances of films were tested by a UV–vis spectrometer (UV-2550, Shimadzu, Kyoto, Japan) with a wavelength range of 200–1000 nm and a Carl Zeiss (Axio Observer A1, Jena, Germany) inverted microscope equipped with crossed polarizers (POM), respectively. The cross-sectional structure was observed by scanning electron microscopy (SEM), which was conducted by JSM-7600F (JEOL, Tokyo, Japan) at an accelerating voltage of 3 kV for magnification of ×2000. All the films were coated with a layer of gold before observation. The chemical structural information of the films was performed by a Fourier-transformed Nicolet 6700 infrared spectrophotometer (FTIR, Thermo Fisher Scientific, Waltham, MA, USA) equipped with an ATR accessory (ATR-FTIR) which had a scanning range of 800–4000 cm^−1^ and an X-ray photoelectron spectrometer (XPS, Shimadzu AXIS UltraDLD, Japan) to detect the elements of C, O, and Zn. The crystal information was collected by a Rigku (Tokyo, Japan) Ultima IV X-ray diffractometer (XRD) with Cu Kα-radiation (λ = 0.15419 nm). The obtained XRD patterns were fitted using the pseudo-Voigt peak shape with Maud Rietveld software (Version 2.7) [34]. The crystallinity index (CrI) of the samples was calculated based on the fitted results via the equation below:

(1) $$\mathrm{CrI}=\frac{A_{crystal}}{A_{crystal}+A_{amorph}}\times 100\%$$

## 4. Conclusions

In the present work, we successfully fabricated highly reflective nanocomposite films with an obvious iridescent effect via evaporation-induced self-assembly (EISA) by mixing cellulose nanocrystals (CNC), xylose, and ZnO NPs at various content ratios. The presence of chiral nematic structures in the films resulted in optical iridescence and birefringence. It was found that the iridescent properties can be tuned by the addition of ZnO NPs. Specifically, intercalation of ZnO NPs led to a redshifted film color due to an enlarged pitch for the chiral helix structure. However, when the content of ZnO NPs exceeded a certain threshold, iridescence disappearance in the M5 film can be observed, accompanied by a weakened polarization phenomenon and reduced light transmissibility. This was confirmed by SEM observation, which revealed vanished cholesteric structures. Moreover, the nanocomposite films exhibited environmental responsiveness, particularly towards humidity and ethanol exposure. As humidity increased or ethanol was added, the films underwent a color redshift. We envision that the construction approach offers new opportunities for designing and fabricating functional materials, including those employed in environmental protection and responsiveness, wearable devices, and food packaging.

## Figures and Tables

**Table 1:** Composition of the prepared films with different ratios of CNC, Xylose, and ZnO NPs.

Film	CNC (g)	Xylose (g)	ZnO NPs (g)
M0	0.1	0.02	0
M1	0.1	0.02	0.001
M2	0.1	0.02	0.005
M3	0.1	0.02	0.01
M4	0.1	0.02	0.015
M5	0.1	0.02	0.02

## Data Availability

All data generated or analyzed during this study are included in this published article and its additional files.
